# Pediatric Dialysis: From Acute Kidney Injury to Chronic Renal Replacement Therapies: Challenges and Perspectives in Resource-Limited Countries

**DOI:** 10.3390/diseases14030111

**Published:** 2026-03-19

**Authors:** Djilali Batouche, Djamila Djahida Batouche, Zoheir Zakaria Addou, Souhila Fatima Bouchama, Rabia Okbani, Siham Simerabet, Nadia Faiza Benatta, Soulef Saadi-Ouslim, Miloud Lahmer

**Affiliations:** 1 Freelance Clinical Research Multihealth, 75000 Paris, France; 2 Université Oran1, Oran 31000, Algeria; batouche.djamila@univ-oran1.dz (D.D.B.);; 3 Laboratoire de recherche Enseignement Et Recherche En Maladies Émergentes Et Ré Émergentes, Oran 31000, Algeria; 4 Laboratoire de recherche Surveillance des infections liées aux soins à Oran, Oran 31000, Algeria; 5 Département de Psychologie, Université Oran 2 Ahmed Benbella, Oran 31000, Algeria; 6 Centre de Recherche en Anthropologie Sociale et Culturelle, Oran 31000, Algeria

**Keywords:** pediatric acute kidney injury, chronic kidney disease, renal replacement therapy, peritoneal dialysis, continuous renal replacement therapy, pediatric kidney transplantation, resource-limited settings, Maghreb, Algeria, health disparities

## Abstract

**Background**: Pediatric kidney failure, whether acute or chronic, constitutes a major public health issue because of its impact on survival, linear growth, neurocognitive development, and long-term quality of life. While high-income countries have markedly improved outcomes through early diagnosis, advanced dialysis technologies, and kidney transplantation, management remains limited in low- and middle-income countries, particularly in the Maghreb region. **Objective**: This review aims to provide an updated synthesis of pediatric kidney failure, with emphasis on renal replacement therapy modalities and the specific challenges encountered in resource-limited contexts, particularly in Algeria. **Methods and Content**: We successively address the pathophysiological and clinical bases of pediatric acute kidney injury and chronic kidney disease, followed by a discussion of available therapeutic strategies: peritoneal dialysis, intermittent hemodialysis, continuous renal replacement therapy, and pediatric kidney transplantation. Particular attention is given to organizational constraints, actual availability of modalities, limited access to consumables and immunosuppressive therapies, and the specificities of pediatric kidney care in the Maghreb region in comparison with international recommendations. **Perspectives**: Improving outcomes for children with kidney failure in Maghreb countries requires a multidimensional approach integrating early screening, strengthening peritoneal dialysis programs, structured development of pediatric kidney transplantation, and enhanced regional and international collaboration. Reinforcing local research capacity and participation in international registries are essential steps toward reducing disparities in care and adapting global guidelines to local realities.

## 1. Introduction

Kidney failure in children represents a complex condition with both immediate and long-term consequences, associated with significant morbidity and mortality, as well as lasting effects on linear growth, neurocognitive development, and quality of life in adulthood [1,2]. Unlike adults, pediatric kidney diseases occur in a developing organism, making management particularly delicate and requiring a specialized multidisciplinary approach.

Acute kidney injury (AKI) in children constitutes a frequent medical emergency in hospital settings, particularly in pediatric intensive care units (PICUs). Its prevalence varies widely depending on the context, reaching up to 30–50% among children admitted to intensive care units in high-income countries [3,4]. In resource-limited settings, the true incidence is probably underestimated due to delayed diagnosis, lack of biological monitoring tools, and the absence of reliable national registries [5]. Etiologies also differ, with a predominance of infectious, hypovolemic, and nephrotoxic causes in low- and middle-income countries [6].

Chronic kidney disease (CKD) in children, although rare, remains a severe condition, predominantly caused by congenital anomalies of the kidney and urinary tract (CAKUT), which account for more than 50% of cases in most pediatric series [1,7]. In Maghreb countries, diagnosis is often established at an advanced stage due to insufficient antenatal screening, irregular neonatal follow-up, and delayed referral to specialized centers [8].

Kidney failure in children is not merely a clinical challenge but a profound socio-economic burden. In resource-limited settings, the management of End-Stage Kidney Disease (ESKD) raises significant ethical dilemmas regarding the allocation of scarce resources. Clinicians often face ‘rationing’ decisions choosing which child receives life-saving dialysis when machines or consumables are limited.

A critical yet overlooked pillar in reducing this burden is primary pediatric screening. Early detection through routine monitoring of growth parameters (weight, height, and BMI) using standardized growth charts is essential. Evidence suggests that subtle deviations in growth velocity often precede clinical symptoms of renal dysfunction. Furthermore, systematic blood pressure measurement and urinalysis (to detect proteinuria or hematuria) at the primary care level could prevent a significant portion of ‘emergency’ dialysis initiations, which are currently the norm in the Maghreb region.

This situation promotes accelerated progression toward end-stage kidney disease (ESKD), with major impact on survival and functional prognosis.

Renal replacement therapies (RRT) represent a cornerstone in the management of advanced kidney failure in children. International recommendations, particularly those from KDIGO and IPNA, advocate for individualized therapeutic strategies according to age, weight, hemodynamic status, and available resources [9,10,11]. Peritoneal dialysis is widely considered the modality of choice in infants and young children, whereas hemodialysis and continuous techniques are reserved for specific clinical situations [12].

However, in resource-limited countries, particularly in North Africa, implementation of these recommendations faces numerous constraints: limited availability of consumables, unequal access to pediatric-specific devices, insufficient specialized training, and major organizational challenges [13,14]. These limitations strongly influence therapeutic choices and partly explain the disparities in outcomes observed between different regions of the world.

Pediatric kidney transplantation remains the gold-standard treatment for end-stage kidney disease, offering the best survival and quality-of-life outcomes [15]. Nevertheless, in the Maghreb context, its development remains limited and relies mainly on living-related donation, in the absence of structured deceased donor programs and in the presence of persistent legal, cultural, and organizational barriers [13,16].

To our knowledge, few reviews have specifically addressed the continuum from pediatric AKI to kidney replacement therapies within the context of Maghreb health systems. This review aims to provide an updated synthesis of pediatric kidney failure management, confronting international recommendations from KDIGO, ISPD, and IPNA with the practical challenges encountered in Algeria. We address the transition from AKI to CKD, the ethical dilemmas of resource allocation, and the strategic importance of developing national registries to bridge the disparity in care. The objective is to identify the main barriers to optimal management and to propose perspectives adapted to resource-limited settings.

**Figure 1:**
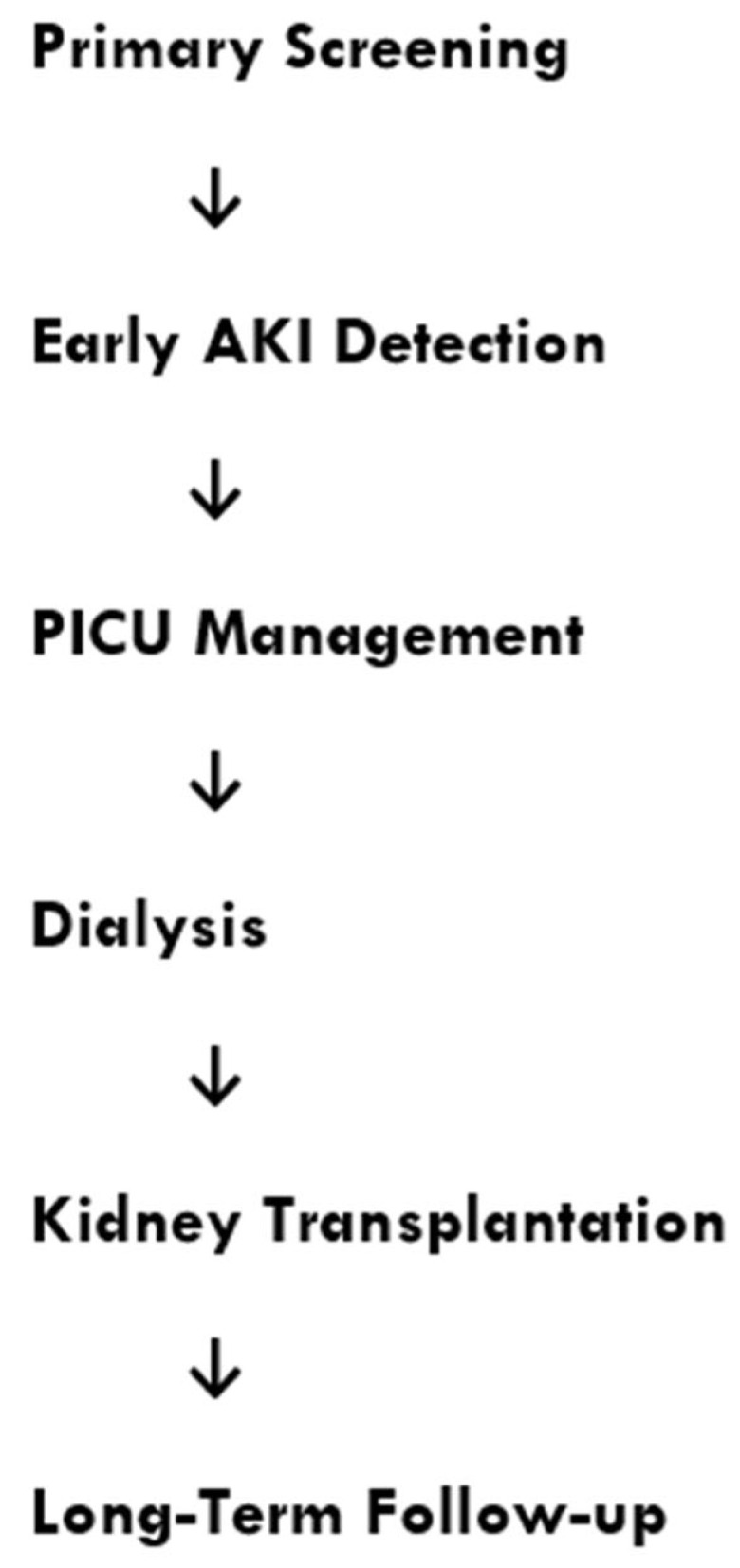
Integrated Pediatric Kidney Care Pathway in Resource-Limited Settings. This schematic representation illustrates the continuum of pediatric kidney care in resource-limited settings. Early identification of kidney injury through primary screening and timely diagnosis of acute kidney injury (AKI) are essential to prevent disease progression. Management in pediatric intensive care units aims to stabilize patients and initiate appropriate renal support when required. Dialysis modalities, including peritoneal dialysis and hemodialysis, constitute critical therapeutic options when renal function cannot be restored. In selected patients with end-stage kidney disease, kidney transplantation remains the optimal long-term treatment. Continuous follow-up is essential to monitor renal function, prevent complications, and improve long-term outcomes. This integrated approach highlights the importance of early detection, access to renal replacement therapies, and structured follow-up to optimize pediatric kidney care in low-resource environments.

The continuum of pediatric kidney care, from early detection of kidney injury to renal replacement therapies and long-term follow-up, is summarized in Figure 1.

## 2. Acute Kidney Injury in Children

### 2.1. Definition and Epidemiology of Acute Kidney Injury

Acute kidney injury (AKI) in children is defined as a sudden impairment of kidney function characterized by an increase in serum creatinine and/or a decrease in urine output, according to the standardized KDIGO criteria [17]. Table 1 shows the diagnostic criteria for acute kidney injury according to pRIFLE and KDIGO.

In pediatrics, AKI is a frequent hospital event, particularly in intensive care settings, where it is associated with significantly increased mortality, prolonged hospital stay, and a higher risk of progression to chronic kidney disease [18,19].

Data from high-income countries report an incidence of AKI reaching 30–50% among children admitted to pediatric intensive care units [20].

In contrast, in resource-limited settings, particularly in North Africa, the true incidence remains poorly documented, mainly due to underdiagnosis, delayed referral to specialized care, and the absence of structured national registries [21,22]. This underestimation likely masks a substantial disease burden, with patients often presenting at more advanced and severe stages.

### 2.2. Etiologies and Specific Features in Resource-Limited Settings

The causes of pediatric AKI vary according to age and geographic context. In Africa, the dominant etiologies (Table 1) include severe infections (sepsis, acute dehydration), hypovolemic shock, postoperative complications, and exposure to nephrotoxic drugs [21,22]. Pre-renal causes remain largely predominant, often associated with delayed initial management.

The following table (Table 2) summarizes the predominant causes.

In low- and middle-income countries (LMICs), the epidemiology of pediatric acute kidney injury (AKI) differs significantly from that reported in high-income settings. While cardiac surgery and drug-related nephrotoxicity are leading causes in industrialized countries [3,4], community-acquired and infection-related AKI predominates in Africa and the Maghreb region [21,22].

Across African pediatric series, prerenal causes account for approximately 40–60% of AKI cases, most frequently related to severe dehydration secondary to acute gastroenteritis, septic shock, and hypovolemia [21,22]. Intrinsic renal causes represent 30–40% of cases and include acute tubular necrosis secondary to sepsis, post-infectious glomerulonephritis, and Nephrotoxins [21]. Post-renal causes are less common, generally accounting for less than 10% of pediatric AKI cases and are mainly related to obstructive uropathies such as posterior urethral valves or lithiasis [22].

In North African cohorts, including Algerian hospital-based studies, hemolytic–uremic syndrome remains a significant cause of intrinsic AKI, with reported incidences varying between 5% and 15% of AKI cases depending on the center. In sub-Saharan Africa, sepsis-related AKI may represent up to 35–45% of cases admitted to pediatric hospitals [21].

Locally in Oran, in pediatric intensive care units, AKI frequently occurs in the context of secondary systemic renal aggression, often associated with multiorgan failure, as demonstrated by Batouche et al. in a recent study conducted in critically ill children [23].

However, as noted by Olowu et al. [24], the outcomes of AKI in sub-Saharan Africa and North Africa are significantly hindered by delayed referral and limited access to basic diagnostic tools like Point-of-Care Testing (POCT) for electrolytes.

The International Society of Nephrology’s “0 by 25” initiative emphasizes that most AKI deaths in LMICs are preventable through early identification and rapid fluid resuscitation.

### 2.3. Diagnosis and Severity Stratification

KDIGO criteria currently represent the reference standard for diagnosing and staging AKI in children [17]. However, strict implementation in daily practice may be limited in low-resource settings due to restricted access to repeated laboratory measurements and precise urine output monitoring.

In this context, early identification of high-risk children relies primarily on clinical evaluation, assessment of risk factors (sepsis, major surgery, nephrotoxic exposure), and careful monitoring of hemodynamic and urinary parameters.

International initiatives such as the “0by25” program of the International Society of Nephrology emphasize the importance of simple and applicable strategies aimed at preventing avoidable deaths related to AKI, particularly in resource-limited countries [25].

### 2.4. Initial Management and Field Limitations

Management of pediatric AKI is based on etiological treatment, optimization of renal perfusion, and prevention of secondary kidney injury. International recommendations emphasize cautious fluid management, avoiding both hypovolemia and fluid overload, both of which are detrimental to kidney and other organ function [26].

In daily practice in the Maghreb and across Africa, this approach often faces organizational constraints: limited access to functional ultrasound, difficulty in dynamic assessment of fluid responsiveness, and delayed initiation of renal replacement therapy. These limitations favor progression toward advanced AKI stages, requiring dialysis under sometimes suboptimal conditions [27,28].

The management of pediatric AKI in resource-limited settings is a cornerstone of Pediatric Intensive Care unit in ORAN Algeria. Unlike the longitudinal follow-up provided by nephrologists, the intensivist must intervene during the ‘golden hours’ of renal aggression. In Algerian PICUs, the clinical reality is a high volume of patients presenting with Acute-on-Chronic Kidney Disease (ACKD) children whose previously unknown CKD is unmasked by an intercurrent stressor such as dehydration or infection.

Furthermore, the intensivist must navigate AKI in the context of multi-organ dysfunction syndrome (MODS), where renal failure is a byproduct of septic shock, post-operative complications, or polytrauma. In these high-acuity scenarios, the decision to initiate RRT is driven by hemodynamic necessity rather than purely biochemical markers.

### 2.5. Real-World Practice in Algeria: Confronting International Recommendations

International guidelines, particularly KDIGO 2012 for AKI, ISPD recommendations for pediatric peritoneal dialysis, and IPNA guidelines for pediatric kidney transplantation, represent the standard of care in high-income countries. However, their application in Algeria remains partial and highly dependent on local resources.

In our pediatric intensive care unit practice in Oran, AKI diagnosis still relies largely on pediatric RIFLE (pRIFLE) criteria, mainly due to their simplicity and long-standing integration into local protocols. Nephrologists, however, refer to KDIGO criteria, which constitute the current international reference.

Strict implementation of KDIGO criteria faces several constraints:

• Limited access to repeated serum creatinine measurements.
• Absence of early biomarkers (NGAL, cystatin C).
• Difficulties in accurate urine output monitoring outside intensive care units.

Thus, unlike countries with national registries and automated laboratory alert systems, AKI diagnosis in Algeria remains frequently clinical and delayed, influencing disease severity at presentation.

Published local data remain scarce. Our studies in pediatric intensive care (ScienceDirect) show that AKI in Oran predominantly occurs in the context of sepsis, hypovolemic shock, or multiorgan failure [23], aligning with African data but contrasting with Western countries where cardiac surgery and iatrogenic causes predominate.

### 2.6. Renal Replacement Modalities: The Technological Divide

In cases of pediatric acute kidney injury (AKI), the indication for renal replacement therapy (RRT) is based on well-established clinical and biological criteria such as refractory fluid overload, severe electrolyte disturbances, persistent metabolic acidosis, or marked elevation of uremic toxins [29,30].

Available renal replacement therapy modalities include:

• Intermittent hemodialysis (HD).
• Peritoneal dialysis (PD).
• Continuous renal replacement therapy (CRRT).

The choice of modality depends on several factors, including the patient’s hemodynamic status, age, weight, underlying condition, and the availability of technical resources and expertise. Peritoneal dialysis remains widely used in low-resource settings due to its simplicity, lower cost, and minimal infrastructure requirements. In contrast, continuous renal replacement therapy is preferred in hemodynamically unstable critically ill children, as it allows for gradual fluid and solute removal. Intermittent hemodialysis, although effective, may be less well tolerated in unstable patients due to rapid fluid shifts.

This disparity in access to advanced technologies highlights a significant global inequity in the management of pediatric AKI, particularly between high-income and low- to middle-income countries.

In high-income countries, CRRT is standard in hemodynamically unstable children. However, in most Maghreb centers, CRRT availability remains extremely limited.

In practice, international recommendations favor continuous renal replacement therapy (CRRT) in hemodynamically unstable children, particularly in the context of sepsis or multiorgan failure [31]. These techniques allow gradual solute clearance and better fluid balance control, due to their ability to provide slower and more continuous removal of fluids and solutes, which generally improves hemodynamic tolerance compared with conventional intermittent hemodialysis [30,31,32,33].

However, implementation of CRRT in children remains extremely limited in low- and middle-income countries because of the high cost of equipment, the need for complex anticoagulation, and insufficient specialized training. Financial, logistical, and human resource constraints represent major barriers to the adoption and sustainability of CRRT programs in these settings [34,35].

In several African and Maghreb countries, particularly in hospital centers such as those in Oran, Algeria, availability of continuous techniques remains very restricted. The absence of local hemofiltration or continuous hemodiafiltration equipment and trained personnel often necessitates repeated sessions of intermittent hemodialysis. Although intermittent hemodialysis may be less well tolerated from a hemodynamic standpoint in unstable children, it frequently represents the only realistic option in these contexts.

Optimization strategies, such as increasing dialysis frequency (e.g., daily or closely spaced sessions), have been proposed to improve tolerance and reduce hemodynamic fluctuations compared with conventional weekly schedules [36]. Although largely derived from adult and pediatric data, this approach supports the concept that greater dialysis frequency may reduce instability and improve metabolic control when access to advanced continuous technologies is limited [36].

Recent technological advances have introduced innovative devices adapted for neonates and small infants. Menon et al. described the development of a novel ultrafiltration device enabling kidney replacement therapy in neonates and infants with very low extracorporeal blood volumes and low blood flow rates, allowing precise ultrafiltration control [37]. The CARPEDIEM system and the NIDUS platform, initially developed in France and later implemented in other high-income countries, have demonstrated safety and feasibility in infants with AKI, opening new perspectives for early pediatric nephrology care [37,38].

In Oran (Algeria) and other university centers, clinicians must often adapt intermittent hemodialysis (IHD) or peritoneal dialysis (PD) to achieve similar goals, despite the higher risk of hemodynamic fluctuations.

Peritoneal dialysis (PD) remains a simple, cost-effective alternative suitable for many pediatric situations, particularly in very young children or where continuous techniques are unavailable [39,40,41]. However, its use may be limited by infection risk and shortages of consumables.

Peritoneal dialysis remains the most feasible and cost-effective modality for pediatric AKI in resource-limited countries. It requires no vascular access or complex anticoagulation. However, its success is contingent on the availability of pediatric-sized catheters and sterile dialysis fluids. Frequent shortages of these consumables often force clinicians to use suboptimal “manual” systems, which increases the risk of peritonitis and technical failure.

Table 3 summarizes the advantages and disadvantages of peritoneal dialysis compared with other RRT modalities [40].

In pediatric or intensive care units where peritoneal dialysis is difficult to implement, or where appropriate pediatric vascular access devices are lacking, intermittent hemodialysis remains, despite its limitations, a necessary modality of renal replacement therapy, especially in low birth weight infants or in cases of difficult vascular access [42]. These disparities are further illustrated in Table 4, which compares pediatric kidney care across different healthcare settings.

**Table 4:** summarizes Pediatric Kidney Care in High-Income vs. Resource-Limited Settings.

Aspect	High-Income Countries	Resource-Limited Countries
Early diagnosis	systematic screening	often delayed
CRRT availability	widespread	limited
Peritoneal dialysis	structured programs	underutilized
Transplantation	living + deceased donor	mainly living donor
Registries	national registries	fragmented data

### 2.7. Progression from Acute Kidney Injury to Chronic Kidney Disease in Children

**Figure 2:**
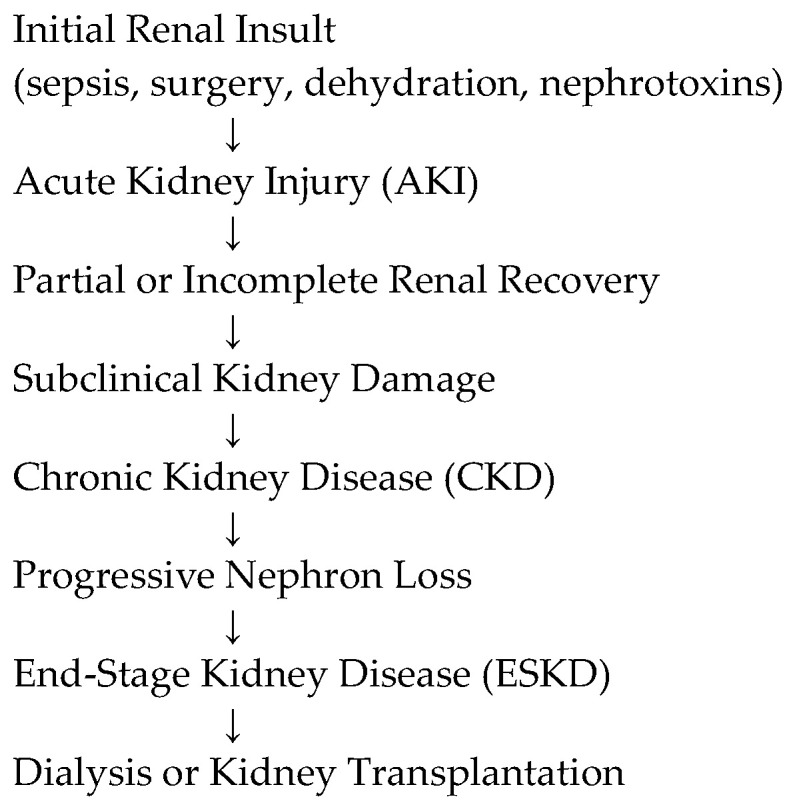
AKI–CKD Progression in Children.

Growing evidence suggests that acute kidney injury in childhood may contribute to the development of chronic kidney disease later in life. This continuum is illustrated in Figure 2.

This figure illustrates the continuum between acute kidney injury (AKI) and chronic kidney disease (CKD) in pediatric patients. Following an initial renal insult, such as sepsis, major surgery, dehydration, or exposure to nephrotoxic medications, children may develop AKI of varying severity. Although renal function may appear to recover clinically, incomplete structural or functional recovery may persist at the nephron level. This subclinical kidney damage can progressively lead to chronic kidney disease, characterized by gradual nephron loss and long-term deterioration of renal function. Without appropriate monitoring and preventive strategies, CKD may evolve toward end-stage kidney disease (ESKD), requiring renal replacement therapies such as dialysis or kidney transplantation. This continuum highlights the importance of early detection of AKI, careful follow-up after apparent recovery, and implementation of preventive measures to limit long-term renal complications in children.

## 3. Chronic Kidney Disease (CKD) in Children

### 3.1. Definition and Epidemiology of Chronic Kidney Disease

Chronic kidney disease (CKD) in children is defined as a progressive and irreversible decline in kidney function lasting more than three months, associated with structural or functional abnormalities of the kidneys, according to international KDIGO criteria [43]. Although less frequent than in adults, pediatric CKD represents a particularly severe condition due to its impact on growth, pubertal development, and long-term survival [44,45,46,47].

In high-income countries, the incidence of pediatric CKD is estimated at 7–12 cases per million children, with a steadily increasing prevalence due to improved survival [7].

In Maghreb countries, particularly Algeria and sub-Saharan Africa, epidemiological data remain fragmented because of the absence of structured national registries. Nevertheless, hospital-based series suggest a similar predominance of CAKUT, often diagnosed at advanced stages [48,49]. Delayed diagnosis is linked to insufficient antenatal Screening, irregular neonatal follow-up, and delayed referral to pediatric nephrology centers, thereby promoting rapid progression to advanced stages of disease.

### 3.2. Causes, Clinical Consequences, and Progression

The main causes of pediatric CKD in the Maghreb include:

• Congenital anomalies of the kidney and urinary tract (CAKUT).
• Hereditary nephropathies.
• Chronic glomerular diseases.

Chronic glomerular diseases include steroid-resistant nephrotic syndrome and lupus nephritis, which represent the leading progressive glomerular causes of CKD in children.

IgA vasculitis-associated nephritis is comparatively less frequent as a cause of end-stage kidney disease (ESKD), with progression occurring in a minority of severe cases [48,49,50].

International registry data indicate that glomerular diseases account for approximately 10–20% of pediatric ESKD, with steroid-resistant nephrotic syndrome and lupus nephritis representing the majority within this category [7,48].

Congenital anomalies of the kidney and urinary tract (CAKUT) represent the leading cause of pediatric CKD, accounting for more than 50% of cases in most international pediatric registries [48,49,50,51]. Recent clinical data confirm that CAKUT remains predominant even in contemporary cohorts, representing the majority of CKD cases diagnosed over the past decade [7].

Pediatric CKD is associated with multiple systemic complications, including growth retardation, nutritional disorders, anemia, disturbances of calcium-phosphate metabolism, and early cardiovascular complications [52,53,54,55,56]. These complications are often more severe in resource-limited settings, where access to adjunctive therapies (erythropoietin, vitamin supplementation, phosphate binders) remains inconsistent.

The clinical correlation between renal function and linear growth is one of the most reliable yet underutilized diagnostic indicators in primary care. In the Algerian school health system, the systematic tracking of height and weight must be viewed as a formal screening for renal health. A growth velocity that falls below the 3rd percentile, or a crossing of two major percentile lines downward, should be interpreted as a potential clinical marker for occult Chronic Kidney Disease (CKD). This ‘growth signal’ often manifests months before biochemical markers like elevated serum creatinine become apparent, especially in cases of Congenital Anomalies of the Kidney and Urinary Tract (CAKUT)

The proposed strategic framework for Algeria relies on a tiered referral system. At the primary level, school medicine acts as the filter through urine dipsticks and blood pressure checks. The second tier involves regional pediatric units capable of managing conservative treatments (nutrition and blood pressure). The final tier consists of university excellence centers dedicated to specialized renal replacement therapies and transplantation. This structured hierarchy aims to decentralize care while concentrating high-tech resources where they are most effective, ensuring a continuum of care from the classroom to the transplant suite.”

Progression to end-stage kidney disease is influenced by several factors, including initial disease severity, quality of nephrological follow-up, and access to renal replacement therapy [7,48].

Historically, in many Maghreb and African countries, transition from advanced CKD to renal replacement therapy is frequently unplanned, with late initiation of dialysis under emergency conditions [57,58].

### 3.3. Conservative Management, Preparation for Renal Replacement Therapy

International recommendations emphasize the importance of early conservative management aimed at slowing CKD progression, optimizing growth, and preparing children for potential renal replacement therapy [59,60,61]. This management includes blood pressure control, correction of metabolic abnormalities, and specialized nutritional follow-up.

In resource-limited settings, the “Nutritional Growth Gap” is a major hurdle. And the protein–energy wasting (PEW) in children with CKD is significantly more severe in developing countries due to the high cost of specialized renal supplements.

In Algeria, clinicians must focus on optimizing caloric intake using locally available resources while strictly monitoring the calcium-phosphate balance. The absence of consistent access to recombinant human growth hormone (rhGH) means that nutritional optimization is the only tool available to combat the short stature that affects over 40% of our CKD population.

To avoid progression to advanced stages of kidney disease decompensation, a unique strength of the Algerian healthcare system lies in the School Health Program. This institutionalized network provides a valuable platform for what can be termed “preventive nephrology.” Through systematic clinical screening, school health practitioners are able to detect early and often asymptomatic markers of renal risk, such as hypertension, proteinuria, and impaired growth, thereby enabling earlier referral and intervention.

Early identification of kidney disorders in children remains a critical component of preventing progression to advanced kidney disease. Routine pediatric screening plays an essential role in this process. Regular monitoring of growth parameters, including weight, height, and body mass index using standardized growth curves, may reveal subtle signs of chronic illness. In addition, systematic measurement of blood pressure during pediatric consultations allows early detection of hypertension, which may reflect underlying renal pathology. Simple laboratory assessments, such as urine dipstick testing for proteinuria or hematuria and evaluation of renal function when clinically indicated, can further facilitate early diagnosis. Strengthening these basic pediatric screening strategies is particularly important in resource-limited settings, where delayed diagnosis frequently contributes to the progression from acute kidney injury to chronic kidney disease and ultimately end-stage kidney disease.

The importance of this screening cannot be overstated. As an ambassador for AKI and CKD in Algeria, it is evident that school-based detection is often the only time a child from a rural or low-income background receives a formal health screening. However, the efficacy of this program relies on a robust referral system. If a positive dipstick test at school does not lead to a pediatric nephrology consultation within weeks, the opportunity for early intervention is lost.

#### Preparation for Renal Replacement Therapy

Preparation for RRT represents a crucial step in the care pathway of children with advanced CKD. Recommendations advocate early preparation including family education, selection of the most appropriate dialysis modality, and planning of vascular or peritoneal access [62,63,64].

In daily practice in Algeria, such preparation is frequently insufficient due to late diagnosis and limited human and material resources in certain hospitals. despite preventive medicine in schools.

### 3.4. Real Access to Renal Replacement Modalities

KDIGO guidelines recommend CRRT in cases of hemodynamic instability. In European or North American reference centers, availability exceeds 80–90% of pediatric intensive care units.

In Algeria and more broadly in the Maghreb, CRRT remains very limited due to:

• Inconsistent availability of generators.
• High cost of disposable kits.
• Limited access to regional citrate anticoagulation.
• Restricted specialized training.

In practice, in the absence of CRRT, closely spaced intermittent hemodialysis sessions are preferred despite sometimes imperfect hemodynamic tolerance. This local adaptation represents a therapeutic compromise dictated by available resources.

Peritoneal dialysis, recommended by ISPD as the modality of choice in infants, remains underutilized despite its lower cost and technical feasibility. Identified barriers include:

• Periodic shortages of consumables.
• Lack of appropriately sized pediatric catheters.
• Insufficient specific training in peripheral centers.

Thus, although international standards are known and theoretically adopted, their implementation remains conditioned by logistical and economic constraints.

## 4. Pediatric Kidney Transplantation: Current Situation and Perspectives in the Maghreb

Pediatric kidney transplantation is recognized by IPNA as the treatment of choice for end-stage kidney disease in children.

In Algeria, the experience reported by Benziane and Boutennoune [16] describes 32 pediatric transplantations performed between 2007 and 2014, mainly from living-related donors, with satisfactory short- and medium-term outcomes. These data demonstrate the technical feasibility of transplantation in centers with adequate infrastructure.

Husain SA [65] examines access to kidney transplants for children and young adults with congenital anomalies of the kidney and urinary tract (CAKUT). It highlights disparities in transplant eligibility and outcomes compared to other patient populations. The authors emphasize the need for improved guidelines and policies to enhance access and support for this vulnerable group. Overall, the study calls for increased awareness and targeted interventions to address these inequities.

However, the vast majority of transplants rely on living-related donation. The first deceased donor transplantations were reported by the Blida team (Si-Ahmed et al.) [66], but their development remains limited.

Rekhif [67] identifies several major obstacles to the development of deceased organ donation in Algeria:

• Limited societal awareness.
• Absence of structured national coordination.
• Administrative constraints.
• Difficulties in identifying brain-dead donors.

Regional analyses published in Access to kidney transplantation for patients with end-stage renal failure in Maghreb countries highlight additional issues [13]:

• Organizational heterogeneity.
• Absence of pediatric national registries.
• Significant regional disparities in access.

In the Maghreb, despite the technical expertise available in university hospitals like those in Oran or Algiers, the number of pediatric transplants remains low.

The primary bottleneck is the heavy reliance on living-related donors. While this provides excellent short-term results, it is insufficient to meet the growing demand. The development of a National Deceased Donor Program is an ethical and clinical necessity. Such a program requires not only surgical infrastructure but also a profound shift in societal awareness and a transparent legal framework to manage organ allocation.

In Morocco, data from Souilmi et al. (2014) [68] confirm the predominance of living donation (>90% of transplants), with a limited number of pediatric recipients. Same observation made by [69].

Unlike European countries where registries such as ERA-EDTA and ESPN allow structured longitudinal follow-up, Maghreb data remain fragmented, limiting long-term evaluation.

## 5. Systemic Constraints in Pediatric Kidney Care

Analysis of regional literature (Elhalimi & Batouche, 2022 [70]; Meguellati et al., 2024 [71]) highlights:

• Absence of a national pediatric registry.
• Unequal access to modern immunosuppressive agents [72].
• Limited immunological monitoring.
• Insufficient specialized training [73].
• Excessive centralization of activity in a few university centers.
• Adolescent Transition Programs: Ensuring that teenagers do not lose their grafts due to non-adherence during the transition to adult care a period known globally for high graft loss rates These constraints explain the gap between international recommendations and clinical reality [74,75].

## 6. Conclusions and Strategic Perspectives

The management of pediatric kidney diseases in Algeria and across the Maghreb region clearly illustrates the persistent gap between international recommendations and real-world applicability in resource-limited settings. Although KDIGO, ISPD, and IPNA standards are well recognized and increasingly integrated into specialized training programs, their full implementation remains constrained by structural, economic, and organizational limitations.

Our field experience in Oran highlights a pragmatic coexistence of diagnostic and therapeutic frameworks: pediatric intensivists frequently rely on pRIFLE criteria due to feasibility, while nephrologists apply KDIGO recommendations whenever biological and technical resources allow. This dual approach does not reflect inconsistency but rather adaptive clinical practice within constrained environments.

Despite documented technical feasibility of pediatric kidney transplantation in selected Algerian centers, sustainable development remains hindered by the absence of a structured national deceased donor program, insufficient coordination mechanisms, and lack of prospective national registries. Consequently, long-term outcome evaluation and strategic planning remain limited.

More broadly, pediatric renal care in the Maghreb is characterized by:

• Late diagnosis of CKD and unplanned initiation of dialysis.
• Limited access to continuous renal replacement therapy.
• Underutilization of peritoneal dialysis despite its suitability for low-resource contexts.
• Unequal access to modern immunosuppressive agents.
• Fragmented epidemiological data.

Addressing these challenges requires a multidimensional and realistic strategy tailored to local capacities rather than simple replication of high-income country models.

Priority actions should include:

1. Establishment of a national pediatric kidney disease registry to generate reliable epidemiological data and guide health policy.
2. Structured expansion of peritoneal dialysis programs, particularly for infants and small children.
3. Progressive development of CRRT expertise through targeted training and resource optimization.
4. Securing continuous access to dialysis consumables and essential immunosuppressive therapies.
5. Development of a coordinated national organ donation and transplantation network, including gradual strengthening of deceased donor programs.
6. Integration into international collaborative networks (IPNA, ESPN) to enhance research participation and benchmarking.

Ultimately, improving outcomes for children with kidney disease in the Maghreb will require a stepwise and context-adapted approach combining local clinical expertise, health system strengthening, and progressive alignment with international standards. Sustainable progress depends not only on technological acquisition but also on structured governance, workforce training, and regional cooperation.

In conclusion, as an ambassador for kidney health in Algeria, the goal of this synthesis is to advocate for a tiered referral system. By strengthening the link between school-based detection and specialized university excellence centers (CHUs), we can ensure that every child regardless of their socioeconomic background receives timely intervention.

The challenges are structural, but the clinical expertise and the institutional frameworks already exist in Algeria to significantly alter the survival and quality of life for the next generation of pediatric patients.

## Figures and Tables

**Table 1:** Diagnostic Criteria for AKI Across pRIFLE, and KDIGO Definitions.

Staging	**p-RIFLE**		**KDIGO**	
	Risk	Estimated creatinine clearance decrease by 25%	Stage 1	Increase in serum creatinine 1.5–1.9 times from baseline Or Increase creatinine 0.3 mg/dL
	Injury	Estimated creatinine clearance decrease by 50%	Stage 2	Increase in serum creatinine 2.0–2.9 times from baseline
	Failure	Estimated creatinine clearance decrease by 75% Or Estimated creatinine clearance < 35 mL/min/1.73 m^2^	Stage 3	Increase in serum creatinine 3 times from baseline Or Serum creatinine 4 mg/dL Or Initiation of renal replacement therapy

This table illustrates the two primary diagnostic frameworks for pediatric AKI. While the KDIGO criteria (based on serum creatinine and urine output) represent the current international reference, the pRIFLE criteria (Risk, Injury, Failure) remain widely used in Algerian PICUs due to their pragmatic clinical utility. The figure highlights how pRIFLE allows for a rapid assessment even when the child’s baseline creatinine is unknown, which is common in emergency admissions in the Maghreb region.

**Table 2:** Main Etiologies of Pediatric AKI in Low- and Middle-Income Countries.

Category	Most Frequent Causes in LMICs	Approximate Proportion
Prerenal AKI	Severe dehydration (gastroenteritis), septic shock, hypovolemia	40–60%
Intrinsic AKI	Acute tubular necrosis (sepsis-related), post-infectious glomerulonephritis, hemolytic–uremic syndrome	30–50%
Postrenal AKI	Obstructive uropathies (posterior urethral valves, stones)	<10%

Data derived from African and North African pediatric hospital series. This table contrasts the epidemiological profiles of pediatric Acute Kidney Injury (AKI) between high-income regions and the Maghreb. It highlights the predominance of community-acquired causes such as severe dehydration, septic shock, and Hemolytic-Uremic Syndrome (HUS). The high percentage of prerenal causes (40–60%) underscores the critical window for early fluid resuscitation. In the absence of advanced biomarkers like NGAL, these etiologies remain the primary focus for triage and initial management in Algerian pediatric intensive care units.

**Table 3:** Advantages and disadvantages of renal replacement therapies in pediatric AKI {IntechOpen 2024}.

Modality	Advantages	Disadvantages
Peritoneal dialysis (PD)	- Easy to perform, especially in resource-limited settings. - No need for complex vascular access. - Better hemodynamic tolerance. - Preferred in infants and small children.	- Lower clearance compared to extracorporeal methods. - Risk of peritonitis. - Contraindicated in recent abdominal surgery or peritoneal anomalies.
Continuous venovenous hemofiltration (CVVH/CRRT)	- Precise fluid and solute control. - Efficient clearance of small and middle molecules. - Well tolerated in hemodynamically unstable patients. - Suitable for prolonged use.	- Requires central venous access. - Expensive and technically demanding. - Requires specialized staff and continuous monitoring.
Intermittent hemodialysis (IHD)	- Rapid clearance of solutes and toxins. - Short treatment sessions. - Widely available and standardized protocols.	- Poor tolerance in unstable children (risk of hypotension). - Technically difficult in small infants. - Rapid shifts in fluid and electrolytes.

A comparative analysis of the three primary RRT modalities used in pediatric intensive care. Peritoneal Dialysis (PD) is presented as the most feasible and cost-effective method for resource-limited settings, offering hemodynamic stability for neonates without the need for vascular access. Intermittent Hemodialysis (IHD) provides rapid clearance but poses significant risks of hypotension in unstable children. Continuous Renal Replacement Therapy (CRRT) is the gold standard for hemodynamic instability; however, its implementation in Algeria is hindered by the high cost of specialized generators and consumables, as well as the technical complexity of regional citrate anticoagulation.

## Data Availability

The review did not generate any data that could be analysed; it was a scientific review based on existing data.
